# P-192. Evaluating the Association of Unnecessary Antibiotic Use with Hospital-Onset *Clostridioides difficile* Infection at a New York City Hospital

**DOI:** 10.1093/ofid/ofae631.396

**Published:** 2025-01-29

**Authors:** Amie John, Antoinette M Acbo, Kelsie Cowman, Yi Guo, Priya Nori, Gregory D Weston

**Affiliations:** Montefiore Medical Center, Bronx, New York; Rutgers University / Jersey Shore University Medical Center , Neptune City, NJ; Montefiore Medical Center, Bronx, New York; Montefiore Medical Center, Bronx, New York; Montefiore Health System, Bronx, NY; Montefiore Medical Center and Albert Einstein College of Medicine, Bronx, NY

## Abstract

**Background:**

Hospital Onset-*Clostridioides difficile* infection (HO-CDI) is defined as collection of a positive *C. difficile* lab result on or after day 4 of a patient’s hospital admission. Reduction of HO-CDI remains a multidisciplinary effort. Prior studies have demonstrated frequent inappropriate antibiotic use among patients with CDI. The objective of this study is to evaluate unnecessary antibiotic use in patients with cases of National Healthcare Safety Network (NHSN)-defined HO-CDI compared to non-CDI controls.

**Figure:**
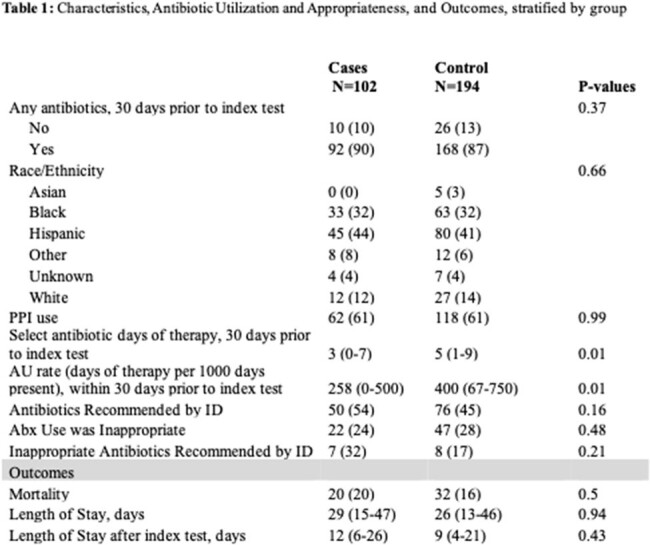

**Methods:**

This case-control study included inpatient adult patients, from 1/1/23 – 2/28/24. HO-CDI cases were selected from standard institutional review. Non-CDI controls were selected based on hospital admission and negative CDI test sent on or later than day 4 of their admission. Cases and controls were matched 2:1 using patient location and index tests within the same month. Antibiotic use (AU) rate of cefepime, ciprofloxacin, levofloxacin, ceftriaxone, and piperacillin-tazobactam 30 days prior to index test was calculated using NHSN definitions. Primary exposure of interest was inappropriate antibiotic use. Outcomes including hospital length of stay (LOS), all-cause mortality, and subsequent recurrent CDI were recorded. A secondary analysis assessed the relationship between race/ethnicity, inappropriate antibiotic use and HO-CDI.

**Figure:**
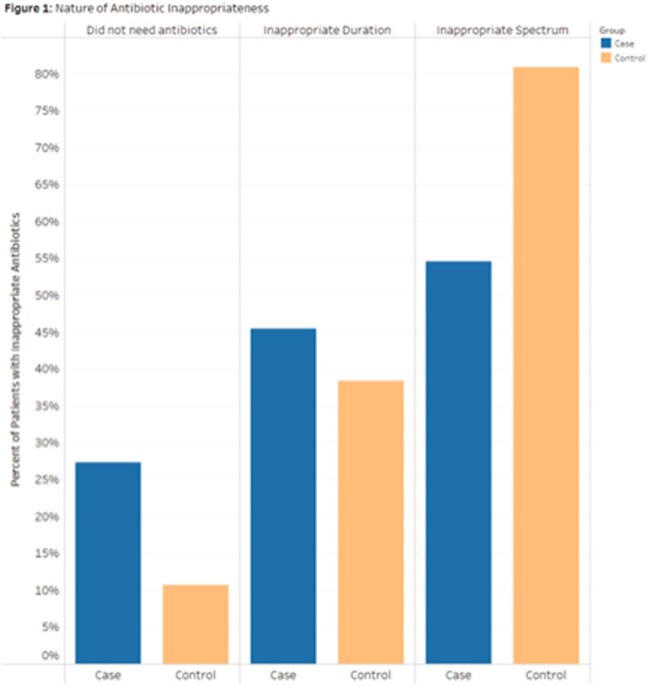

**Results:**

Two hundred ninety-six patients were included. AU rate was greater in controls compared to HO-CDI cases (p = 0.01) (Table 1). Inappropriate AU was observed in 24% of cases and 28% of controls (p = 0.48). The most common category of inappropriate AU was inappropriate spectrum, 55% in cases and 81% in controls (Fig 1). There were no statistically significant differences in outcomes.

**Conclusion:**

HO-CDI cases did not have greater exposure to antibiotic therapy and did not have a higher proportion of inappropriate antibiotic use compared to patients who tested negative for *C. difficile*. This demonstrates an opportunity to improve antibiotic use, but does not show an association between inappropriate antibiotic use and *C. difficile* infection.

**Disclosures:**

**All Authors**: No reported disclosures

